# Optimisation of surface expression using the AIDA autotransporter

**DOI:** 10.1186/1475-2859-10-72

**Published:** 2011-09-14

**Authors:** Martin Gustavsson, Emma Bäcklund, Gen Larsson

**Affiliations:** 1 School of Biotechnology, Division of Bioprocess Technology, Royal Institute of Technology, KTH, SE 106 91 Stockholm, Sweden

## Abstract

**Background:**

Bacterial surface display is of interest in many applications, including live vaccine development, screening of protein libraries and the development of whole cell biocatalysts. The goal of this work was to understand which parameters result in production of large quantities of cells that at the same time express desired levels of the chosen protein on the cell surface. For this purpose, staphylococcal protein Z was expressed using the AIDA autotransporter in *Escherichia coli*.

**Results:**

The use of an OmpT-negative *E. coli *mutant resulted in successful expression of the protein on the surface, while a clear degradation pattern was found in the wild type. The expression in the mutant resulted also in a more narrow distribution of the surface-anchored protein within the population. Medium optimisation showed that minimal medium with glucose gave more than four times as high expression as LB-medium. Glucose limited fed-batch was used to increase the cell productivity and the highest protein levels were found at the highest feed rates. A maintained high surface expression up to cell dry weights of 18 g l^-1 ^could also be achieved by repeated glucose additions in batch cultivation where production was eventually reduced by low oxygen levels. In spite of this, the distribution in the bacterial population of the surface protein was narrower using the batch technique.

**Conclusions:**

A number of parameters in recombinant protein production were seen to influence the surface expression of the model protein with respect both to the productivity and to the display on the individual cell. The choice of medium and the cell design to remove proteolytic cleavage were however the most important. Both fed-batch and batch processing can be successfully used, but prolonged batch processing is probably only possible if the chosen strain has a low acetic acid production.

## Background

Bacterial surface display of proteins is a topic that has gathered a lot of research interest since its discovery. This technology is of interest for several applications, including live vaccine development, bioremediation through adsorption to expressed binder proteins, library screening and the development of whole cell biocatalysts [1,2].

Protein expression in Gram-negative bacteria generally offers several advantages over Gram-positive, mainly due to the knowledge and understanding of growth and production in *Escherichia coli*. However, surface expression in this host is theoretically more complicated since transport of the expressed proteins must take place over two cell membranes compared to only one in Gram-positive strains. Additionally, there is a lack of protein transporters in *E. coli *laboratory strains and few proteins can therefore be found outside the cell. The discovery of the type V autotransporter family in pathogenic strains and its successful transplantation into commercial strains has however opened up new possibilities with regard to surface expression [3].

Autotransport of a protein to the outside of a cell is given by a vector-based mechanism present in a wide family of Gram-negative microorganisms. The vector consists of three major parts: an N-terminal signal sequence for passage over the inner membrane, a passenger protein to be exposed on the cell surface and a C-terminal β-barrel anchored in the outer membrane. The native function of autotransporters is the export of virulence factors in pathogenic Gram-negative bacteria, and the natural passengers include for example proteases, adhesins and toxins [4]. Ultimately, the fate of the passenger protein is either to remain anchored to the cell surface or to be cleaved off and released to the surrounding environment. The *E. coli *Adhesin Involved in Diffuse Adherence (AIDA-I) [5] is an example of an autotransporter that has been used for surface display of enzymes [6], enzyme inhibitors [7], potential antigens for vaccine development [8] and several other applications.

Successful use of surface expression technology for production of live vaccines or biocatalysis requires the production of large quantities of cells while maintaining a desired level of surface expression. The understanding of which were the main factors to influence the development of such a process was therefore the goal of this work. The strategy included the investigation of the influence of the host strain, the choice of medium and the medium effects on induction of the AIDA wild type promoter, and the effect of the chosen process technique. Since effects relating to the protein structure was not the task of this work, a protein with a positive excretion potential was chosen. Protein Z, the synthetic B domain of staphylococcal protein A [9], was selected since it is a naturally surface-anchored protein in *Staphylococci *but also because it is able to bind to the F_c _region of IgG which makes it easy to detect. Expression takes place from the native promoter for AIDA-I, *aidA *[10]. This, together with the low plasmid copy number, should ensure that the cellular burden of the overexpression system is also minimised.

## Materials and methods

### Bacterial strain and medium

*Escherichia coli *K12 strain 0:17 (sup^+^, F^-^) [11] and 0:17ΔOmpT, both provided by Professor Leif Isaksson, were used in this work. Cultivations were performed in minimal salt medium and Luria-Bertani (LB) medium. The composition of the minimal salt medium was (per litre): 7 g (NH_4_)_2_SO_4_, 1.6 g KH_2_PO_4_, 6.6 g Na_2_HPO_4 _·2H_2_O and 0.5 g (NH_4_)_2_-H-Citrate. Sterile glucose solution (500 g l^-1^) was added to the minimal medium to a concentration of 10 g l^-1 ^unless otherwise stated. In addition, the minimal medium was supplemented with 1 ml l^-1 ^trace element solution and 1 ml l^-1 ^MgSO_4 _solution (1M), both sterile filtered through 0.2 μm filters (Sartorius) The trace element solution consisted of (per litre): 0.5 g CaCl_2 _·2H_2_O, 16.7 g FeCl_3 _·6H_2_O, 0.18 g ZnSO_4 _·7H_2_O, 0.16 g CuSO_4_·5H_2_O, 0.15 g MnSO_4 _·4H_2_O, 0.18 g CoCl_2 _·6H_2_O, 20.1 g Na-EDTA. In the high-density bioreactor cultivations, additional trace element solution and MgSO_4 _was added for every 20 OD units. The feed solution used during fed-batch cultivations consisted of 30% (w/w) glucose in distilled water.

The LB medium contained (per litre): 10 g tryptone, 5 g yeast extract and 10 g NaCl. In some cultivations the LB medium was additionally supplemented with glucose to a concentration of 10 g l^-1^, as noted. The cultures were supplemented with 100 mg ampicillin per litre of medium to ensure plasmid stability.

### Construction of vector pMK90-Z

**Figure 1:**
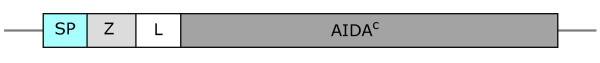
**Surface expression vector**. Components are shown to approximate and relative sizes. The construct contains the native signal sequence for AIDA (SP), the protein Z passenger (Z), a linker region (L) and the C-terminal β-barrel domain (AIDA^c^) of AIDA.

Plasmid pMK90, a derivative of the low copy-number plasmid pBR322, expresses a recombinant AIDA-transported protein and has been described previously [12]. The vector contains the native 49 aa AIDA signal peptide, a linker consisting of a multiple cloning site of 24 amino acids combined with the C-terminal 54 amino acids of the native AIDA passenger, followed by the C-terminal 440 amino acid AIDA β-barrel. The expression of the fusion protein is under the control of the native *aidA *promoter located between the aah and aidA genes, which has previously been shown to be dependent on the genetic background of the expression strain [10]. In this vector, the cleavage site for releasing the passenger protein from the surface, which is present in the AIDA wild type vector, has been removed. The vector was digested with XmaI and dephosphorylated with Antarctic phosphatase. A portion of plasmid pK4ZZ-cutinase-wt [13] containing protein Z was PCR-amplified using primers EJ1FORZZ and EJ8REVZZ (primer sequences, see below). The PCR product was digested with XmaI and ligated into vector pMK90, previously digested with XmaI. The presence of an insert in the vector was checked by PCR amplification using primers EJ4FORSS and EJ5REVTRANS. The correct orientation of the insert was verified by PCR amplification using primers EJ1FORZZ and EJ5REVTRANS. DNA sequencing to determine the sequence of the construct was performed using primers EJ4FORSS and EJ10REVTRANS and an ABI Prism 3700 DNA analyser (Applied Biosystems). The final vector was given the name pMK90-Z and is shown in Figure 1. Cloning enzymes were purchased from New England Biolabs and used according to the suppliers' recommendations. Oligonucleotides used for DNA sequencing and vector constructions were ordered from MWG Biotech.

#### Primers

EJ1FORZZ-5'- CGA GCG TAC CCG GGA CGT AGA CAA CAA ATT CAA CAA AGA AC

EJ4FORSS-5'- AAT AAG GCC TAC AGT ATC ATA TGG

EJ5REVTRANS-5'- CAG CAT GGA ATT TAT AAA TAT CCC

EJ8REVZZ-5'- GCG ACA GTA GAC CCG GGA TTT CGG CGC CTG AGC ATC ATT TAG

EJ10REVTRANS-5'- CTG TAG CAG AGA GCA GCT TTG C

### Cultivation

Unless otherwise stated, all cultivations where performed at 37°C in minimal salt medium. Small-scale cultivations were performed using baffled shake flasks and a working volume of 10% to ensure sufficient aeration. Flasks were placed in a heated incubator (Infors Minitron) with shaking (180 RPM).

Fed-batch cultivations were performed in a six-parallel bioreactor system (Greta, Belach Bioteknik AB, Sweden). Culture volumes were 800 ml and the reactors were inoculated with 5% of the working volume from an overnight shake flask culture, giving a starting OD_600 _of approximately 0.2. Glucose (30% w/w) was fed at an exponentially increasing rate in order to keep the cells growing with a constant specific growth rate (h^-1^). The feed rate at time t (F_t_, l h^-1^) was given from a mass balance of glucose omitting the low concentration of glucose in the medium (equation 1). The exponent μ (h^-1^), equal to the specific growth rate, is given a desired value according to the actual experiment, x (g) is the cell mass when the batch phase ends, V (l) is the medium volume, s_in _(g l^-1^) is the feed glucose concentration and Y_xs _(g g^-1^) is the cell dry weight yield per gram of glucose.

(1) $$F_{t}=\frac{\mu \cdot x\cdot V}{Y_{xs}\cdot s_{in}}$$

The initial batch phase was run with 3 g l^-1 ^glucose with a yield of 0.5, giving an approximate cell mass of 1.5 g l^-1 ^at the start of the glucose feed. Three feed exponents were chosen as: 0.1 h^-1^, 0.2 h^-1 ^and 0.4 h^-1^, respectively. Each experiment was run in duplicate.

Bioreactor batch cultivations were performed in a 15 l stirred tank bioreactor (Belach Bioteknik AB, Sweden) with a working volume of 10 l. Inoculation was done from an overnight shake flask culture in exponential phase. To reach a high cell mass without the use of fed-batch technology, repeated batch-wise additions of glucose (50% w/w) to a concentration of 10 g l^-1 ^were made when the previously added glucose was consumed.

The pH was kept constant at 7.0 during the bioreactor cultivations through automatic titration with 25% and 12.5% (v/v) ammonium hydroxide for the 15 l and six-parallel reactors, respectively. The dissolved oxygen tension (DOT) was kept above 30% at all times by manually varying the stirrer speed. The airflow was initially 0.1 VVM and was then increased stepwise to 1 VVM during the cultivation. Sterile antifoam was manually added when necessary to avoid excess foaming.

### Analysis

#### Cell mass

The increase in cell mass was monitored by measuring the optical density at 600 nm (OD_600_) using a photometer (Novaspec, Pharmacia Biotech). The samples during the cultivation were diluted to approximately the same OD_600 _using saline (0.9% NaCl) and the actual OD_600 _was obtained by multiplying by the dilution factor. For cultivations in the 15 l reactor, cell dry weight was monitored by centrifugation (10 minutes, 4500 rpm) of 5 ml samples in triplicate in dried, pre-weighed glass tubes. The resulting pellets were washed with saline, dried overnight at 105°C and allowed to cool in a desiccator. The cell dry weights were obtained as the weight increase of the pre-weighed tubes.

#### Acetic acid

Samples of acetic acid (HAc) formation were taken by filtering the supernatants obtained from the dry weight samples through a 0.2 μm syringe filter (Sartorius). The acetic acid concentrations were determined using a commercially available kit (Boehringer-Mannheim no. 148261) in accordance with the instructions of the manufacturer.

#### Outer membrane protein

Outer membrane protein (OMP) fractions were isolated as described in the literature [14]. The protein solutions obtained were separated on a 10% SDS-PAGE gel (NuPAGE, Invitrogen) and blotted to nitrocellulose membranes (Bio-Rad). The membranes were blocked overnight with phosphate buffered saline (PBS, 9g l^-1 ^NaCl, 0.21g l^-1 ^KH_2_PO_4_, 0.726 g l^-1 ^Na_2_HPO_4_, pH 7.4) containing 5% milk powder. After blocking, the membranes were incubated with rabbit serum against the C-terminal domain of AIDA (AIDA^c^) followed by the addition of goat anti-rabbit IgG linked to alkaline phosphatase. Finally the membranes were developed by the addition of SIGMA FAST BCIP/NBT (5-Bromo-4-chloro-3-indolyl phosphate/Nitro blue tetrazolium, Sigma) and the developed membranes were scanned.

#### Surface expression

Samples taken for monitoring the surface expression of protein Z were diluted to an OD_600 _of approximately 1, mixed with equal parts of sterile glycerol solution (50% v/v) and frozen at -70°C. Glycerol was added to protect the cells from damage due to ice crystal formation. A sample was taken from a cultivation and stored for a month to understand the analytical stability during this procedure. The surface expression was then compared with that of a fresh sample of the same OD_600 _from an identical cultivation, and no difference was found (data not shown).

On the day of analysis, 50 μl samples were thawed and washed with followed 800 μl PBS followed by centrifugation (10 minutes, 4500 rpm, 4°C) in a tabletop centrifuge for Eppendorf tubes. After centrifugation, the cells were labelled using the antibody-binding properties of protein Z as follows. The cell pellets were resuspended in 100 μl of 100 nM biotinylated polyclonal human IgG1 and incubated at room temperature for 1 h with end-over-end mixing. After washing the sample with 100 μl PBS, the tubes were covered in foil and the cells were incubated on ice with Streptavidin-AlexaFluor488 (Invitrogen) diluted 1:1000 in PBS. After a final wash with 100 μl PBS, the sample was resuspended in 200 μl ice cold PBS and analysed using a FACS VantageSE flow cytometer (BD Bioscience). Fluorescence was compared to that of a negative control (*E. coli *containing the empty surface expression vector pMK90).

#### Amino acids

To determine the amino acid concentration in the cultivation medium, samples were taken by centrifugation of cell suspensions followed by filtration through a 0.2 μm syringe filter and finally storage at -20°C. On the day of analysis, any proteins present in the samples were precipitated by the addition of 40 μl trichloroacetic acid (TCA, 0.612 M) per 500 μl of sample, vortexing for 10 s and incubation for 20 min at room temperature. After incubation, the samples were centrifuged (13 000 rpm, 10 min). The supernatants were then sent for commercial HPLC analysis.

## Results

### Strain-dependent expression of protein Z

**Figure 2:**
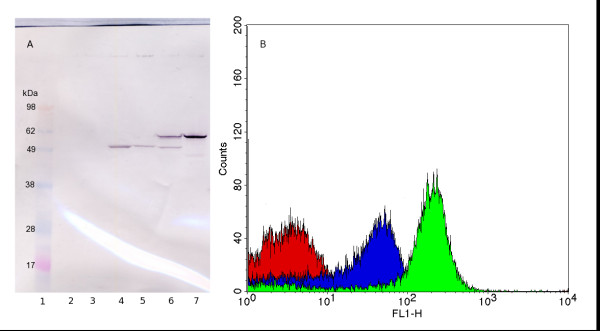
**Strain dependent expression of protein Z**. Comparison of surface expression of protein Z in the wild type and in the OmpT-negative *E. coli *strain 0:17. (A): Western blot of the outer membrane protein fraction, developed using antiserum against AIDA^c^. Lane 1: Marker, Lane 2: 0:17, Lane 3: 0:17ΔOmpT, Lane 4: 0:17 with empty vector, Lane 5: 0:17ΔOmpT with empty vector, Lane 6: 0:17 with vector containing protein Z, Lane 7: 0:17ΔOmpT with vector containing protein Z. (B): Cells analysed by flow cytometry using human IgG linked to AlexaFluor488. Red: 0:17 with empty expression vector, Blue: 0:17 with vector containing protein Z, Green: 0:17ΔOmpT with vector containing protein Z.

A wild-type *E. coli *strain was chosen as the host for the optimisation since the strain grows on simple, well-defined growth media resulting in predictable and reproducible growth and protein production. However, many proteins, including the selected model protein Z, contain potential cleavage sites for the outer membrane protease OmpT, which preferentially cleaves between two consecutive basic amino acids (here Lys_49 _- Lys_50_) [15]. To evaluate the effect of the presence of OmpT in the host cell, the vector was transformed into both the wild type and an OmpT-negative mutant (0:17ΔOmpT). Both strains were grown at 30°C in an attempt to minimize the effect of temperature-dependent OmpT expression in the wild type. The outer membrane protein fractions of the two strains were then isolated and analysed by Western blotting using rabbit antiserum against AIDA^c ^(Figure 2A). Protein Z was successfully expressed in the outer membrane in both the strains, and was detected at the expected size of 62.7 kDa for the AIDA-Z fusion. However, the protein was partly degraded in the wild type, as indicated by a band of approximately the same size as that of the AIDA^c ^reference (55.7 kDa), which is the expected size from OmpT-cleavage of the protein Z passenger. A stronger band for the full-length protein is visible in the sample from the OmpT-negative mutant, indicating that less proteolysis is present. However, proteolysis was not completely abolished, as seen by a band below 49 kDa, indicating a cleavage site in AIDA^c^. This shows that other proteases than OmpT can also degrade the protein, but to a lesser degree.

Following confirmation of the localisation of the AIDA-Z construct in the outer membrane, the correct orientation of protein Z towards the external environment was verified using flow cytometry. Intact cells were labelled using biotinylated human IgG coupled to streptavidin-AlexaFluor488, followed by flow cytometric analysis (Figure 2B). The results confirm that protein Z was successfully expressed on the cell surface and accessible to the medium. In addition, the results show that the surface-expressed protein Z is functional with respect to IgG binding. The fluorescence is markedly reduced in the wild type indicating that a lower amount of functional protein is located on the surface in this strain, supporting the findings from the Western blot. This shows that growing the cells at a lower temperature is not sufficient to remove the proteolysis due to OmpT, and it is clear that the OmpT-negative mutant is needed to maximise the surface expression of protein Z. Finally, the surface expression of protein Z in 0:17ΔOmpT grown in minimal medium at 30 and 37°C was compared using flow cytometry analysis. A higher expression was verified at 37°C (550 fluorescence units per cell, compared to 160 at 30°C) and all subsequent experiments were therefore carried out at this temperature, which is also an advantage during process conditions due to the higher growth rate.

### Expression in stationary phase

**Figure 3:**
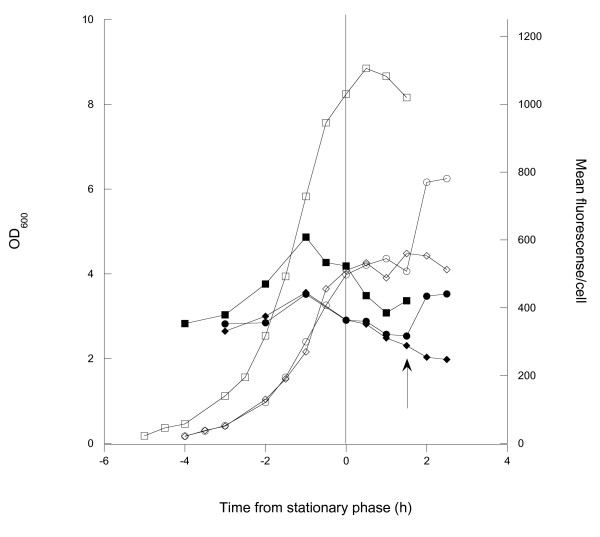
**Effects of carbon starvation on surface expression**. Shake flask cultivations of 0:17ΔOmpT showing growth and surface expression of the Z protein in relation to the entry into stationary phase. Three cultivations are shown: one with 10 g l^-1 ^initial glucose (squares) and two with 3 g l^-1 ^initial glucose (circles and diamonds). Open symbols show cell growth measured by optical density and filled symbols show surface expression measured by flow cytometry. Arrow: a second addition of glucose was made to one of the two flasks with low starting glucose (circles). All curves are normalized to enter stationary phase simultaneously, and the entry into the stationary phase is marked with a vertical line.

Relatively little is known about requirements for induction of the wild type *aidA *promoter. In a recent study by Benz *et al*., a higher expression was found from this promoter when the cells entered the stationary phase, although the reason for entry was not declared. Since no influence of the stationary phase sigma factor encoded by *rpoS *was found in the study by Benz *et al*., we hypothesised that the increased expression could instead result from guanosine tetra phosphate (ppGpp) formation during stringent response, as mediated by the *spoT *gene due to carbon starvation [16]. To test this hypothesis, cells were grown in minimal medium with glucose in shake flasks until all the glucose was consumed. Two flasks were grown with a low glucose concentration (3 g l^-1^) to enter the stationary phase early, and a reference flask was grown with a higher concentration of glucose (10 g l^-1^). Samples for surface expression were taken during the cultivation and analysed using flow cytometry. After 1.5 h in the stationary phase, a second addition of glucose solution to a concentration of 5 g l^-1 ^was made to one of the flasks with low starting glucose concentration, leading to a resumption of growth. Figure 3 shows the surface expression and cell mass as a function of time for the different cultures. The results show that the surface expression per cell increases with time in the lag and log phases, respectively. However, the expression stops when the cells enter the stationary phase. The production resumed when glucose was added to one of the cultures. Thus no increase in expression from the promoter could be seen. This does not rule out the possibility that the expression could be influenced by a stringent response mediated by the *relA *gene, in this case because of entry into the stationary phase due to amino acid starvation.

### Effects of growth medium

**Figure 4:**
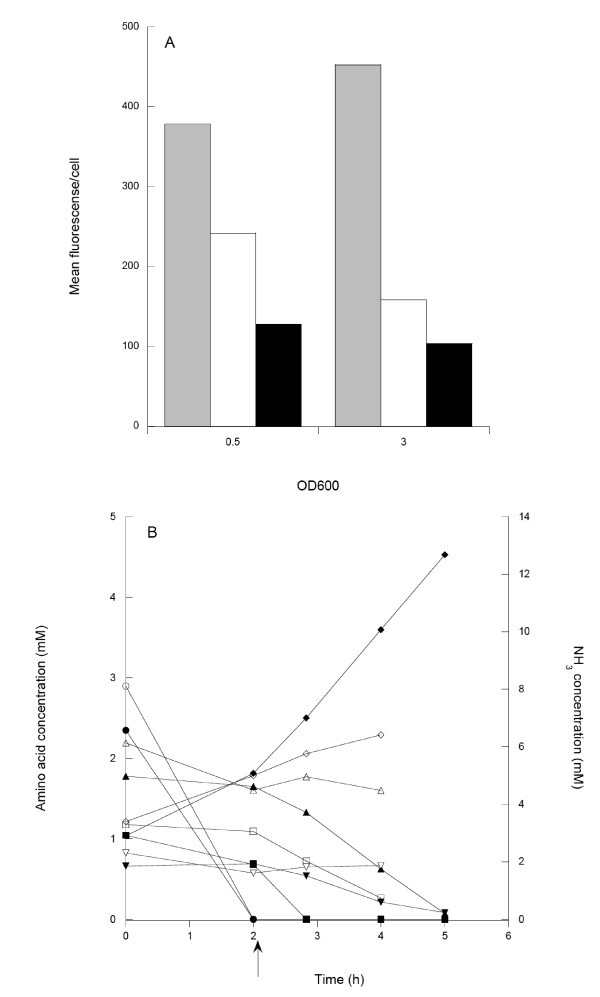
**Effects of growth medium**. (A) Comparison of surface expression of protein Z in minimal medium with glucose (grey), LB medium (white) and LB medium with glucose (black). Samples were taken at OD_600 _= 0.5 and 3. (B) Consumption of amino acids and production of NH_3 _in LB medium (filled symbols) and LB medium with glucose (open symbols) as a function of cultivation time. Legend: Serine (circles), aspartate (squares), threonine (upwards pointing triangle), proline (downwards pointing triangle) and NH_3 _(diamonds). Arrow: depletion of the first amino acid (serine) in the LB cultures.

To test this, 0:17ΔOmpT was grown both in a minimal medium with glucose as carbon source and in a complex LB medium with and without glucose supply. Cells grown in LB medium supplemented with glucose should have a lower consumption of amino acids used in the energy metabolism [17] and would thus not be depleted with respect to selected amino acids as readily as in the simple LB medium. This consumption pattern was verified as shown in Figure 4a, where the energy amino acids were rapidly consumed in the LB medium. Cells growing in LB in the presence of glucose consumed energy amino acids less rapidly, with the exception of serine, as reported previously [17]. The surface expression was highest in the minimal medium with glucose, which was 4.5 times higher than in LB medium with glucose (Figure 4b). In the rich medium, the expression was drastically reduced and was lowest in LB with glucose.

### Effects of growth rate

It was then hypothesized that surface expression might be inversely coupled to the growth rate, since addition of glucose lead to reduced expression. Furthermore, going from minimal medium to LB medium and finally to LB medium with glucose represents an increase in growth rate while the cells produced at progressively lower rates. This suggests that a lower growth rate, but not stretching as far as total depletion, might be a better choice for production.

**Figure 5:**
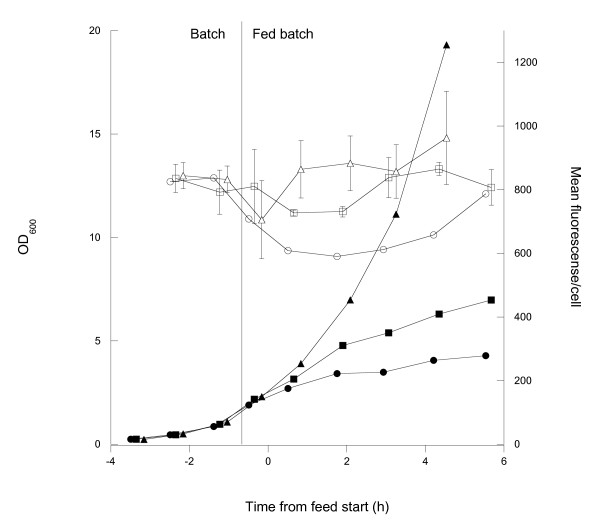
**Fed-batch cultivation**. Progress of cell growth and surface expression before and after feed start (marked with a vertical line). Filled symbols show cell growth and open symbols show surface expression. Legend: μ = 0.1 h^-1 ^(circles), μ = 0.2 h^-1 ^(squares) and μ = 0.4 h^-1 ^(triangles).

Glucose limited fed-batch cultivations performed in minimal medium with three different exponential feed rates (theoretically resulting in growth rates of 0.1, 0.2 and 0.4 h^-1^, respectively) were chosen to support growth. Surface expression was followed both during the initial batch phase and after feed start and growth curves and surface expression are shown in Figure 5. The expression per cell is high but relatively uniform at all feed rates. The surface expression momentarily drops after the feed start at the lower rates with the most marked drop (approximately 10%) being at the lowest feed rate. With time, the surface expression returns to values comparable to those achieved during the batch phase. However, there is a tendency for the expression to be lower at lower growth rates. This is further enhanced if looking at the productivity, which is significantly higher in the fast growing cells due to the cell growth rate and OD reached. The highest feed rate also shows data varying around the batch level value and does not decrease as the other two. At lower feed rates, it is further seen that not only surface expression but also growth is impaired. This indicates that the synthesis and translocation of the AIDA-Z fusion is a burden that also affects growth when glucose is strictly limited.

### Repeated batch

From the experiments reported above, a high growth rate seems to promote high expression. The highest possible growth rates would of course be reached in batch cultivation, but this is generally a concept avoided in commercial production due to the low cell productivity and risk of accumulation of unwanted by-products such as acetic acid.

**Figure 6:**
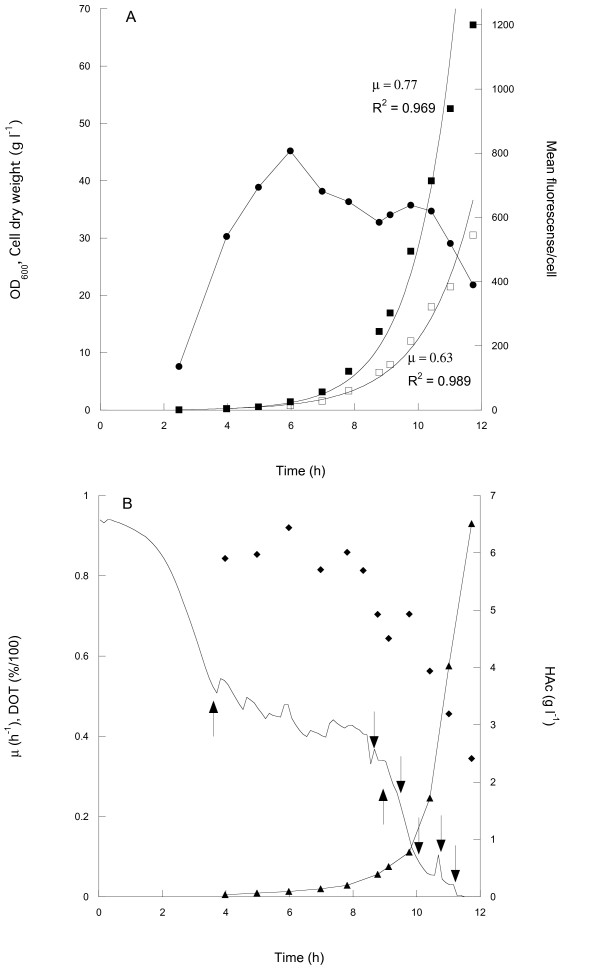
**Batch cultivation with repeated glucose addition**. (A) Cell growth measured as optical density (filled squares) and dry weight (open squares), and surface expression measured by flow cytometry (circles) as a function of cultivation time. Growth curves are fitted to exponential functions to approximate the growth rates (optical density and dry weight). (B) Growth rate (diamonds), acetic acid concentration in the growth medium (triangles) and dissolved oxygen tension (DOT, line) as a function of time. Downward pointing arrows indicate time points for the five additions of glucose. The leftmost upwards pointing arrow indicates the starting point for manual DOT regulation by increasing of the stirring or the air-flow, and the rightmost upwards pointing arrow indicates the point at which the maximum stirrer speed was reached.

To increase the productivity, also in the batch process, repeated glucose additions might be used at least until the reactor oxygen transfer rate is exhausted. Using a total of six additions, a batch process was conducted where a final cell dry weight of approximately 30 g l^-1 ^was reached. Figure 6a shows cell growth, acetic acid accumulation and surface expression during the cultivation. Flow cytometric analysis showed that a high surface expression could be maintained up to cell densities of around 18 g l^-1^, at a point were there was a sharp drop in the expression level. This drop occurred when oxygen limitation lead to mixed acid fermentation as indicated by the sharp increase in acetic acid production at very low DOT values (Figure 6b).

**Figure 7:**
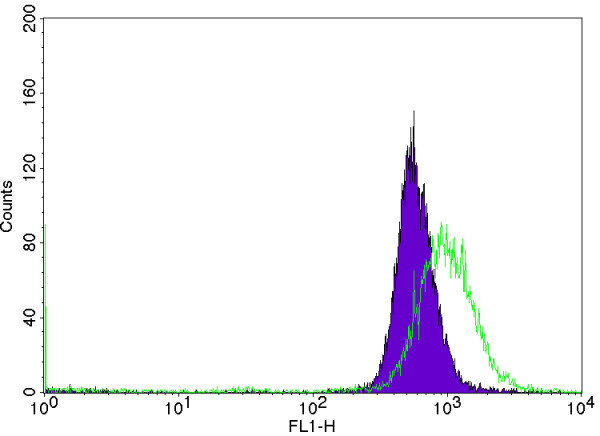
**Typical fluorescence peaks characterising batch and fed-batch expression**. Comparison of fluorescence peak width between a selected but typical fed-batch experiment here from a cultivation at a feed corresponding to a growth rate of 0.4 h^-1 ^(green) and the batch experiment with repeated glucose additions (purple).

The surface expression evaluation for these experiments resulted in the unexpected effect that fluorescence peaks were generally narrower than those in the fed-batch experiment (Figure 7). This indicates less diversity in the surface expression of the bacterial population in batch processing. Comparing the volumetric productivities shows higher values for the batch cultivation than in the highest growth-rate fed-batch (19.8**^.^**10^15 ^fluorescence l^-1^compared to 10.2**^. ^**10^15^), even though the expression per cell is lower in the batch cultivation. This is mainly because of the higher specific growth rate, but also due to the fact that a higher cell density was reached in the batch cultivation.

## Discussion

The transfer of a protein to the outer part of the cell exposes the protein to a range of different challenges specifically in relation to proteolytic degradation. From our different experiments with the AIDA system [8] we draw the conclusion that the critical path is the translocation over the outer membrane and that proteins are often cleaved close to the N-terminus, i.e. in the outer part of the protein with respect to the cell wall. The OmpT cleavage of protein Z could here be relatively easily removed due to knowledge of the substrate specificity and the direct use of this mutant is probably a good advice for a randomly selected protein. For other proteins, in this case selected *Salmonella *epitopes, this has not been as simple [8].

The design of the cultivation medium resulted in a great impact on the surface expression where the minimal medium with glucose as carbon/energy source was a considerably better choice than the other media tested. The constant increase in the growth rate between these media, from minimal to rich media, and the corresponding decrease in protein expression, makes it is tempting to suggest that it is a lower growth rate that causes the better expression levels. It has been found, for many proteins that are difficult for the cell to produce, that this is the case [18]. If these proteins are not immediately degraded they need more time to be produced in an active form or alternatively to be translocated to other compartments before correct folding can take place. In different recombinant protein expression systems, a decrease in the temperature is often used for the reason of giving a lower rate of synthesis and thus a lower growth rate, which can sometimes promote a higher expression level. Even though a higher growth rate in minimal medium gave higher expression levels in our case, it can not be excluded that growth rate may have an influence in rich media such as LB since the cells grow even faster than what can be achieved in minimal medium experiments. There might thus exist an optimum growth rate for maximum expression regarding the present protein and the weak expression system used. A too low carbon supply leads to too low energy levels, and a too high supply of carbon and amino acids leads to overload of the cellular capacity.

Our findings on surface expression in different media correspond well with recent findings of Benz *et al*. [10], who studied the intracellular expression of β-galactosidase under the control of the *aidA *promoter, i.e. the same promoter that was used in the present study. For this intracellular expression system the expression in minimal medium was also better than in a complex medium. The expression difference was approximately the same as that observed here, which supports the idea that the reduced expression in LB medium is rather coupled to unknown effects of promoter control in certain media than to the surface transport process.

The reduced surface expression when entering the stationary phase at carbon/energy starvation indicates that the *aidA *promoter is probably not induced by ppGpp formation by a *spoT*-dependent stringent response. Furthermore, since amino acids were depleted in both LB media, although to different degrees, this should lead ppGpp accumulation due to a *relA*-dependent stringent response, which should lead to an increased expression. Since this did not happen, the results indicate that the *aidA *promoter is not positively affected by the stringent response.

These findings are however to some extent in contrast to the findings of Benz *et al *[10] who found an increased expression from the *aidA *promoter during entry into stationary phase. Their study also verified that there was not a coupling of promoter induction to the general carbon starvation response system (*rpoS*). However, it should be noted that experiments were performed using a complex medium and it cannot be conclusively said which limitation led to stationary phase entry in this study. Thus, the effect observed may be different from the one seen in the present work and could be due to regulatory components of other substrates.

Fed-batch cultivation was initially applied to optimise a simultaneous cell mass and product accumulation. While high expression levels were found with exponential profiles, the establishment of a standard industrial high cell density profile protocol, with exponential followed by constant feed, was not considered as a way to go since this would rapidly lead to very low growth rates which we found unfavourable for expression.

We turned instead to batch cultivation with repeated additions of glucose to increase the cell density. This resulted in comparatively high cell values while the production levels were reasonably stable. The development of a commercial process around this batch concept seems also to result in more stable product values and any undesired variation in the glucose supply, which would negatively influence the production, can be avoided since the glucose concentration is constantly kept at non-limiting levels through batch wise additions (Figure 6).

However, the high cell density values were only reached since the 0:17 *E. coli *strain produces low amounts of acetic acid. The negative effects of acetic acid on growth and recombinant protein production have been shown before [19] and accumulation needs to be avoided. Different strains of *E. coli *are known to produce different amounts of acetic acid even though their growth rate is similar. For 0:17 and another wild type K12 strain, AF1000 (MC4100 relA^+^) the critical acetic acid concentration were growth is impaired is reached at very different levels; around 8 and 3 g l^-1 ^cell dry weight, respectively. The 0:17 cells kept growing until around 20 g l^-1 ^before any severe effect on the growth rate was seen. Eventually, the reactor oxygen transfer rate is approached which can clearly be seen in Figure 6b, as there is a sharp increase in the production of acetic acid due to mixed acid fermentation, which coincides with the DOT values dropping towards 0% air saturation. Up to this point, the repeated batch strategy offers significant advantages in ease of handling. In the large production scale, the oxygen transfer capacity will be considerably lower, leading to a lower maximum obtainable cell mass. Thus, the repeated batch is for this reason suboptimal for cultivation on this scale.

The data from flow cytometric analysis (Figure 7) showed that the distribution of surface expression obtained during the extended batch cultivation is narrower than those obtained during the fed-batch cultivations, indicating that the bacterial population is more homogeneous. The peak width is an important quality parameter and with a more homogeneous population a better control of the concentration of expressed protein can be achieved. This effect is not seen in the fed-batch experiment presented in this work, nor has the effect been observed during fed-batch experiments for extended time periods in the same 10-l reactor as was used for the batch cultivation (unpublished data). This behaviour has previously been attributed to differential substrate uptake rates of the cells in a population due to different expression levels of the sugar permeases, leading to different production rates in different cells [20].

## Conclusions

A number of parameters in recombinant protein production were seen to influence the AIDA-based surface expression of the model protein with respect both to the productivity and to the display on the individual cell. This included the choice of cell, the medium, the temperature and the cultivation technique. We found that the medium and the cell design leading to a removal of proteolytic cleavage were however amongst the most important factors and a minimal medium was shown to be highly preferential. Both fed-batch and batch processing can be successfully used, specifically at high glucose uptake rates, but prolonged batch processing is probably only possible using strains with low acetic acid production. This includes the 0:17 K12 strain but some B-strains (e.g. *E. coli *BL21) are most likely equally good candidates.

The conditions of induction of the AIDA wild type promoter remain unclear. Attempts were made to increase the expression by subjecting the cells to conditions well known for promoting a stringent response i.e. with either carbon or amino acid starvation, but no such positive effects were observed, rather the opposite. These difficulties suggest that in further optimisation experiments it might be better to consider an inducible and tuneable promoter, since it is evident that a current lack of sufficient knowledge of the *aidA *promoter is a limitation when designing a stable process for surface expression.

Except for controlling the productivity, the parameters studied could also be used to influence the amount of protein on each cell in the population, where the batch concept gave the narrowest distribution. However, not all applications of surface expressing cells may require this. For instance, in a whole cell biocatalysis process it may be more important to have a high total enzymatic activity in the population than to have the same activity per cell. For these applications it might be more feasible to use a fed-batch strategy to reach higher cell masses than is possible using a batch approach due to the comparatively rapid exhaust of oxygen. For live vaccines, on the other hand, it might be more important to have a homogeneous distribution, since the dosage needs to be tightly controlled. In this case it seems inadvisable to choose the fed-batch approach It also has to be mentioned that if the population distribution we observe is indeed due to difference in glucose uptake in the cell population, the situation will probably become worse during large-scale processing, as sugar gradients due to mixing will become an additional factor [21].

## Competing interests

The authors declare that they have no competing interests.

## Authors' contributions

MG performed the majority of the expression experiments and wrote the manuscript. EB did the cloning, performed the wild type and mutant experiments and contributed to the manuscript. GL was responsible for the original concept, supervised the work and contributed to the manuscript. All authors have read and approved the manuscript.
